# GPAA1 promotes gastric cancer progression via upregulation of GPI-anchored protein and enhancement of ERBB signalling pathway

**DOI:** 10.1186/s13046-019-1218-8

**Published:** 2019-05-22

**Authors:** Xiao-Xin Zhang, Bo Ni, Qing Li, Li-Peng Hu, Shu-Heng Jiang, Rong-Kun Li, Guang-Ang Tian, Li-Li Zhu, Jun Li, Xue-Li Zhang, Yan-Li Zhang, Xiao-Mei Yang, Qin Yang, Ya-Hui Wang, Chun-Chao Zhu, Zhi-Gang Zhang

**Affiliations:** 1 State Key Laboratory of Oncogenes and Related Genes, Shanghai Cancer Institute, Ren Ji Hospital, School of Medicine, Shanghai Jiao Tong University, Shanghai, 200240 People’s Republic of China; 2 Department of Gastrointestinal Surgery, Ren Ji Hospital, School of Medicine, Shanghai Jiao Tong University, Shanghai, 200217 People’s Republic of China; 3 Shanghai Medical College of Fudan University, Shanghai, 200032 People’s Republic of China; 4 Department of Interventional Radiology, Tongren Hospital, School of Medicine, Shanghai Jiao Tong University, Shanghai, 200336 People’s Republic of China

**Keywords:** Gastric cancer, GPAA1, GPI-anchored proteins, EGFR, ERBB2

## Abstract

**Background:**

Gastric cancer is one of the deadliest malignant tumours, with a high incidence in China, and is regulated by aberrantly overexpressed oncogenes. However, existing therapies are insufficient to meet patients’ needs; thus, the identification of additional therapeutic targets and exploration of the underlying mechanism are urgently needed. GPAA1 is the subunit of the GPI transamidase that transfers the GPI anchor to proteins within the ER. The functional impacts of increased expression levels of GPAA1 in human cancers are not well understood.

**Methods:**

Data mining was performed to determine the pattern of GPAA1 expression and the reason for its overexpression in tumour and adjacent normal tissues. In vitro and in vivo experiments evaluating proliferation and metastasis were performed using cells with stable deletion or overexpression of GPAA1. A tissue microarray established by the Ren Ji Hospital was utilized to analyse the expression profile of GPAA1 and its correlation with prognosis. Western blotting, an in situ proximity ligation assay, and co-immunoprecipitation (co-IP) were performed to reveal the mechanism of GPAA1 in gastric cancer.

**Results:**

GPAA1 was a markedly upregulated oncogene in gastric cancer due to chromosomal amplification. GPAA1 overexpression was confirmed in specimens from the Ren Ji cohort and was associated with ERBB2 expression, predicting unsatisfactory patient outcomes. Aberrantly upregulated GPAA1 dramatically contributed to cancer growth and metastasis in in vitro and in vivo studies. Mechanistically, GPAA1 enhanced the levels of metastasis-associated GPI-anchored proteins to increase tumour metastasis and intensified lipid raft formation, which consequently promoted the interaction between EGFR and ERBB2 as well as downstream pro-proliferative signalling.

**Conclusions:**

GPAA1 facilitates the expression of cancer-related GPI-anchored proteins and supplies a more robust platform—the lipid raft—to promote EGFR-ERBB2 dimerization, which further contributes to tumour growth and metastasis and to cancer progression. GPAA1 could be a promising diagnostic biomarker and therapeutic target for gastric cancer.

**Electronic supplementary material:**

The online version of this article (10.1186/s13046-019-1218-8) contains supplementary material, which is available to authorized users.

## Background

Worldwide, gastric cancer (GC) is the fourth most prevalent malignant tumour and the second most lethal cancer [1]. In 2008, 1 million patients were diagnosed; 74% of these were from East Asia, including 47% in China [2]. Currently, surgery, chemotherapy and targeted therapy, including Erb-B2 receptor tyrosine kinase 2 (ERBB2) and vascular endothelial growth factor receptor (VEGFR) inhibitors, are the effective gastric cancer therapies, but the long-term survival rate remains unsatisfactory [3]. Uncontrolled proliferation and metastasis contribute to the poor prognosis of gastric cancer patients, and a better understanding of the molecular mechanism is crucial for the development of novel treatments.

Cancer is a lethal disease caused by the genetic alterations, including the upregulation of oncogenes and the downregulation of tumour suppressors [4]. Glycosylphosphatidylinositol (GPI)-anchored proteins (GPI-APs) are modified proteins that attach to cell membranes via a GPI anchor, essential to multiple cellular functions, including cell adhesion, metabolism, proliferation and immune regulation; among them, some GPI-APs are responsible for tumorigenesis and progression [5]. Semaphorin 7A (SEMA7A), a GPI-AP, has been found to promote EGFR-tyrosine kinase inhibitor (TKI) resistance in EGFR-mutated lung cancer through activating the mTOR pathway [6]. Prostate stem cell antigen (PSCA), another type of GPI-AP, has been indicated to play an important role in tumorigenesis, proliferation and cell cycle progression via the upregulation of c-Myc expression [7]. In addition, alkaline phosphatase (ALP), glypican-3 (GPC3), and carcinoembryonic antigen (CEA) have been identified as biomarkers of cancers [8–10].

In addition to mediating specific biological functions, GPI-APs contribute to signal transduction through constructing a platform, the lipid raft, to facilitate interactions among different proteins and molecules. The cell membrane is heterogeneous, featuring a variety of distinct components; GPI-APs are some of the most important players, comprising the microdomain of lipid rafts and endowing the cell membrane with flexibility and mobility to mediate protein interactions and signal transduction [11, 12]. The cellular function of lipid rafts includes increasing the concentration of signalling molecules, inducing conformational changes in the membrane, and mediating interactions between receptors and phosphorylation by kinases. For example, Src family members are enriched in raft-like domains, and mitogenic signalling is initiated from several types of surface receptors [13]. Thus, we assumed that genetic mutations, epigenetic alterations, or protein synthesis alterations can not only influence the levels of cancer-associated GPI-APs but also regulate membrane signalling transduction involved in cancer, which fundamentally affects the initiation and development of cancers, including stomach cancer.

The process of GPI-AP synthesis contains several steps, among which the addition of GPI anchors to precursor proteins, which is mediated by the GPI transamidase complex (GPIT), is the most important. To date, five subunits have been identified to comprise the GPI transamidase complex: PIG-T, GPI8, PIG-S, PIG-U and GPI anchor attachment 1 (GPAA1, [14]). Among these components, GPAA1 is responsible for the linkage between the GPI anchor and the protein. A GPAA1 mutant produced complete GPI precursors but cells expressing this mutant failed to express GPI-APs on the cell membrane [15]. GPAA1 has been found to be overexpressed in head and neck squamous cell carcinoma [16], hepatocellular carcinoma [17], breast carcinoma [18], and colorectal carcinoma [19]. However, these results only describe the overexpression of GPAA1 in cancers, while the tumorigenic function and underlying mechanism remain incompletely explored. Coincidently, the gene encoding GPAA1 is located on human chromosome 8q24, a locus with frequent copy number alteration (CNA) closely related to gastric cancer carcinogenesis [20, 21]. Thus, we speculated that GPAA1 expression is highly upregulated in gastric cancer and aimed to investigate its function and molecular mechanism.

Here, we found that GPAA1 is overexpressed in gastric cancer due to chromosomal amplification. In vitro and in vivo experiments showed that GPAA1 promotes the growth and metastasis of gastric cancer. Furthermore, we demonstrated that the mechanism underlying GPAA1 upregulation increased the levels of metastasis-related protein expression and ERBB signalling. Together, these results revealed that GPAA1 might be a promising biomarker and therapeutic target for gastric cancer.

## Materials and methods

### Patients and clinical specimens

The microarray chip included samples from 587 patients with resected primary gastric cancer treated with surgery at Ren Ji Hospital, School of Medicine, Shanghai Jiao Tong University, from 2005 to 2011. All diagnostic data, such as tumour pathology and node and metastasis stage, were gathered based on the American Joint Committee on Cancer (7th edition) guidelines. Before enrolment, all patients provided informed consent, and the study was approved by the Research Ethics Committee of Ren Ji Hospital, School of Medicine, Shanghai Jiao Tong University. The inclusion criteria for this study included the following: 1) an accurate pathologic diagnosis of gastric cancer; 2) the absence of any other kind of solid tumour; 3) the lack of presurgical treatment with chemotherapy, radiotherapy or other anticancer therapies; 4) the acceptance of radical surgery without residual tumour; and 5) the availability of clinicopathological and follow-up data. Because of the loss of follow-up data for 18 patients, only 569 patients were enrolled in the survival analysis, univariate analysis, and multivariate analysis. According to the area and intensity of positive staining, the specimens were scored on a scale with four levels ranging from 0 to 3, which were represented by -, +, ++, and +++ and indicated no expression, weak expression, moderate expression and strong expression, respectively. The scores were independently judged and recorded by two experienced pathologists in a blinded manner.

### Data mining and analysis

Pan-cancer analysis of GPAA1 genomic alterations was performed using the online cBioPortal database (http://www.cbioportal.org/). Copy number analysis of GPAA1 in the The Cancer Genome Atlas (TCGA) stomach carcinoma, oesophageal carcinoma and breast carcinoma databases was conducted via the UCSC Xena website (https://xena.ucsc.edu/welcome-to-ucsc-xena/). GPAA1 gene expression was analysed via microarray gene expression datasets (GSE51575, GSE33335 and GSE29272) downloaded from the Gene Expression Omnibus (GEO). The Kaplan-Meier Plotter online tool (http://kmplot.com/analysis/), a database for analysis of GEO data, was utilized to generate the survival curves. Gene set enrichment analysis (GSEA) was performed by the online tool developed by the Broad Institute (http://software.broadinstitute.org/gsea/index.jsp).

### Immunohistochemistry

The tissue microarray chip and slides were deparaffinized and rehydrated using xylene and a graded series of alcohols. Endogenous peroxidases were removed through incubation with 3% H_2_O_2_ for 20 min at 37 °C. Then, slides were autoclaved in 10 mM sodium citrate (pH 6.0) for 30 min to unmask antigens, washed in TBS (pH 7.4) three times, and incubated with primary antibodies against GPAA1 (1:300, Bioss, bs-13496R), ERBB2 (1:300, abcam, ab16901), p-AKT (1:100, Cell Signaling Technology (CST), #4060), Ki-67 (1:400, Bioss, bs-23105R), MMP2 (1:300, abcam, ab97779), MMP9 (1300, abcam, ab38898), the urokinase receptor (UPAR) (1500, abcam, ab218106) at 4 °C overnight. Slides were incubated with horseradish peroxidase-conjugated secondary antibody for 1 h at room temperature, and signal amplification and detection were conducted using DAB according to the manufacturer’s instructions (CST). Finally, images were acquired with a Nikon microscope.

### Western blot analysis

Cells lysis was performed with total protein extraction buffer (NCM Biotech, Suzhou, China), and tissues were homogenized in T-PER tissue protein extraction reagent (Thermo Fisher Scientific), to which a combination of protease and phosphatase inhibitors was added (NCM Biotech, Suzhou, China). The protein concentration was measured by a BCA Protein Assay Kit (Thermo Fisher Scientific). Cell or tissue lysates (approximately 20 μg of protein) were subjected to SDS-PAGE (8–10%) and transferred to nitrocellulose (NC) membranes. After blocking with 5% fat-free milk, the NC membranes were incubated with different primary antibodies at 4 °C overnight. Antibodies against the following proteins were used: GPAA1 (1:1000, Bioss, bs-13496R), caveolin-1 (1:1000, Abcam, ab2910), AP2B1 (1:1000, Proteintech, 15,690–1-AP), β-actin (1:10000, Abcam, ab5644), EGFR (1:1000, Abcam, ab52894), p-EGFR (Y1068) (1:1000, Abcam, ab40815); ERBB2 (1:1000, Abcam, ab16901); p-ERBB2 (Y877) (1:1000, Abcam, ab47262), p-AKT (S473) (1:1000, CST, #4060), and AKT (1:1000, CST, #4685). After three washes in TBST (pH 7.4), membranes were incubated with species-specific secondary antibodies (Thermo Fisher Scientific) at room temperature for 1 h. Ultimately, protein bands were visualized by an Odyssey imaging system (LI-COR Biosciences, Lincoln, NE, USA).

### Cell culture

Human gastric carcinoma cell lines (MGC-803, AGS, BGC-823, MKN-45, NCI-N87, HGC-27, and SGC-7901) and a normal gastric mucosal epithelial cell line (GES-1) were appropriately maintained at Shanghai Cancer Institute, Ren Ji Hospital, School of Medicine, Shanghai Jiao Tong University. The mouse stomach carcinoma cell line MFC was purchased from the Chinese Academy of Sciences, Shanghai. Cells were cultured in Roswell Park Memorial Institute (RPMI) 1640 medium or Dulbecco’s modified Eagle’s medium (DMEM) supplemented with foetal bovine serum (FBS, 10% (v/v)) and penicillin (100 units/ml) and streptomycin (100 μg/ml). All cells were cultured in a humidified incubator under conditions of 37 °C and 5% CO_2_.

### Quantitative real-time PCR

Cells were washed with PBS (pH 7.4), and total RNA was extracted using TRIzol reagent (Takara, Japan) and reverse transcribed using a PrimeScript RT-PCR kit (Takara, Japan) according to the manufacturer’s instructions. Real-time PCR analysis was performed using SYBR Premix Ex Taq (Takara, Japan) with a 7500 Real-time PCR system (Applied Biosystems, USA). The △△CT method was utilized to calculate the relative expression of specific genes, which was further normalized to the GAPDH mRNA levels. The sequences of the primers are shown in Table 1.

**Table 1:** Sequences of primers used for real-time PCR

Primer	Sequence 5′-3′
GPAA1 Forward	CTCCCGCTTCGTCTCCATC
GPAA1 Reverse	CACTGGCAGGACATAGAGGG
P21 Forward	CGATGGAACTTCGACTTTGTCA
P21 Reverse	GCACAAGGGTACAAGACAGTG
P27 Forward	ATCACAAACCCCTAGAGGGCA
P27 Reverse	GGGTCTGTAGTAGAACTCGGG
CyclinB1 Forward	AATAAGGCGAAGATCAACATGGC
CyclinB1 Reverse	TTTGTTACCAATGTCCCCAAGAG
CyclinD1 Forward	CAATGACCCCGCACGATTTC
CyclinD1 Reverse	CATGGAGGGCGGATTGGAA
CDK4 Forward	TCAGCACAGTTCGTGAGGTG
CDK4 Reverse	GTCCATCAGCCGGACAACAT
CDK6 Forward	CCAGATGGCTCTAACCTCAGT
CDK6 Reverse	AACTTCCACGAAAAAGAGGCTT
GAPDH Forward	CTGGGCTACACTGAGCACC
GAPDH Reverse	AAGTGGTCGTTGAGGGCAATG

### GPAA1 overexpression, knockdown and cell transfection

The Lv-Ctrl, Lv-GPAA1, sh-NC, sh-GPAA1–1, and sh-GPAA1–2 lentiviral constructs were purchased from OBiO Technology Corp., Ltd. (Shanghai). Viruses were transfected at a concentration of 1 × 10^7^–1 × 10^8^/ml. The AGS and SGC-7901 cell lines were transfected with lentivirus expressing sh-NC and sh-GPAA1 because they exhibited the highest expression of GPAA1 among the cell lines, while GPAA1 was overexpressed in MKN-45 and HGC-27 cells according to their limited endogenous expression of GPAA1. When the cell lines were 60–70% confluent, they were transfected with specific lentivirus at a concentration of 1 × 10^6^/ml in the presence of 6 μg/ml polybrene (Sigma-Aldrich, H9268). Cells with stable GPAA1 overexpression or knockdown were cultured in DMEM or RPMI-1640 medium supplemented with 2 μg/ml puromycin (Gibco, A1113802).

### Colony formation and CCK-8 assays

Cells were digested by trypsin (NCM Biotech, Suzhou, China) and resuspended at a density of 1000 cells/well (6-well plate). The medium was replaced with new medium every 4 days. Two weeks later, the formed clones were washed with PBS, fixed with 1% paraformaldehyde solution, and stained overnight with 0.1% crystal violet/40% methanol. After removing the crystal violet by washing, the results were captured by an HP scanner and analysed with ImageJ.

A Cell Counting Kit-8 (CCK-8) assay was performed to examine cell growth. Different types of cells were seeded into 96-well plates at 3000 cells/well, and CCK-8 reagent was added at 0, 24, 48, 72, 96 and 120 h following the instructions of the supplier (Dojindo Molecular Technologies, Japan). After incubating at 37 °C and 5% CO2 for 1 h, cell viability was examined by measuring the absorbance at 450 nm via a Power Wave XS microplate reader (BioTek). The experiments were performed three times following the same protocol.

### Cell cycle assay

Different cell types (sh-Ctrl, sh-GPAA1, Lv-Ctrl, and Lv-GPAA1) were seeded in 6-well plates at a concentration of 3 × 10^6^/ml and were harvested by trypsin treatment. Cells were washed twice with PBS and collected by centrifugation. Cells were fixed with cold 70% ethanol, incubated with RNase A for 1 h at 37 °C and stained with propidium iodide for 20 min at 4 °C. A flow cytometer (FACSCalibur; Becton Dickinson, San Jose, CA, USA) was used to measure the numbers of cells in each phase of the integrated cell cycle 488 nm excitation and 585 nm emission, and the results were analysed by ModFit LT software (Verity Software House, Topsham, USA).

### Immunofluorescence staining

For cell immunofluorescence staining, cells were seeded in immunofluorescence-specialized 12-well plates at a concentration of 1 × 10^5^/ml and incubated at 37 °C overnight. Cells were washed with PBS three times and fixed with 4% polyformaldehyde (15 min), permeabilized with 0.1% Triton X-100 (30 s) and blocked with 10% BSA (60 min) at room temperature. At the interval of each step, cells were washed with PBS 3 times. After blocking, cells were incubated with diluted primary antibodies at different concentrations following the instructions provided by the suppliers (Ki-67, 1:400, bs-23105R; EGFR, 1:300, Abcam, ab52894; and ERBB2, 1:300, Abcam, ab16901) at 4 °C overnight. After washing with PBS three times, cells were incubated with Alexa Fluor 488- and/or 594-conjugated secondary antibody (1:300) at room temperature for 1 h. Finally, cells were covered with an anti-fluorescence-quenching sealing liquid containing DAPI. A Nikon microscope was used to acquire images. Quantitative analysis of fluorescence intensity was performed with ImageJ.

### Tumour cell migration/invasion assays

To determine the migration and invasive abilities of gastric cancer cells with altered expression of GPAA1, wound healing and Transwell assays were conducted. For the wound healing assay, cells were cultured in 6-well plates to 100% confluence. A 10-μl pipette tip was used to generate a wound on the surface of the cells. After washing the cells with PBS, fresh culture medium was added to the 6-well plate. Images of cells were captured and recorded as the first time point. Then, the complete medium was removed and replaced with fresh FBS-free medium, and culture was continued for 48 h. The wound healing status at the 48 h time point was photographed, and the gap closure between the 0 h and 48 h time points was measured using ImageJ software to assess the migratory capability of the cancer cells.

Transwell assays are another effective method to examine the migration and invasive abilities of cells. For the migration assay, cells were harvested and adjusted to a concentration of 5 × 10^4^/ml in 200 μl of serum-free RPMI 1640 medium and seeded in the upper chamber (Millipore PIEP12R48). The lower chambers were filled with 700 μl of medium containing 20% FBS. After 24 h, cells were fixed and stained with 0.1% (w/v) crystal violet. The membrane of the upper chamber was then cleaned, images were taken in three random fields, and the number of cells in each field was counted. The invasive ability was measured via the same general method as the migration ability; the only difference was that the 8-mm pore-size polycarbonate membrane was precoated with a layer of basement membrane matrix (ECMatrix gel) in the invasion assay.

### In vivo metastasis assay

The mouse-derived gastric cancer cell line MFC was transfected with lentivirus containing Luc-plasmid (Plenti-CMV-EGFR-Linker-Luc-PGK-Puro) purchased from OOBIO Biotechnology Co., Ltd. (Shanghai). Luc-transfected MFC (Luc-MFC) cells were also transfected with Lv-GPAA1 lentivirus to upregulate the expression of GPAA1 or with or Lv-Ctrl lentivirus as the control. MFC-Lv-Ctrl and MFC-Lv-GPAA1 cells were injected into the spleens of C57BL/6 N mice (*n* = 5/group) at a concentration of 10^6^ cells/mouse to test the potential capability for liver metastasis. Animals were injected with D-luciferin (150 mg; Promega, catalogue no. P1043), subjected to anaesthesia by isoflurane inhalation, and subjected to luciferin emission imaging to measure the diffusion of cancer cells using an IVIS Spectrum (Caliper Life Sciences) every 5 days. The data were quantified by Living Image software, version 4.5.3. Four weeks later, animals were sacrificed, and an immunohistochemical (IHC) analysis of liver tissue was conducted to evaluate the metastatic ability of the cancer cells.

### Lipid raft extraction

Cells were washed with pre-chilled PBS and lysed for 30 min at 4 °C using 1% Triton X-100 in TNEV buffer (10 mM Tris-HCl, pH 7.5; 5 mM EDTA; 1 mM PMSF; 150 mM NaCl; and 1 mM Na3VO4). A BioVision tissue homogenizer was utilized to conduct cell homogenization. The lysate was separated by centrifugation (200×*g* for 8 min), and the supernatant (400 μl) was added to 400 μl 85% (w/v) sucrose in TNEV buffer. The solution was transferred to a centrifuge tube and overlaid with 35% (w/v) sucrose in TNEV buffer (2.4 ml) and 5% (w/v) sucrose in TNEV buffer (1.4 ml). The samples were centrifuged (200,000×*g*) for 18 h at 4 °C. The fractions were collected from the highest gradient for further western blot analysis.

### In situ proximity ligation assay

An in situ proximity ligation assay (PLA, Olink Bioscience, DUO92007) was performed to evaluate the interaction between two specific proteins. Briefly, AGS and SGC-7901 cells (sh-Ctrl and sh-GPAA1) were plated in immunofluorescence-specialized 12-well plates at a concentration of 1 × 10^5^/ml and incubated at 37 °C overnight. The second day, cells were washed with PBS, fixed with 4% formaldehyde for 20 min at room temperature and permeabilized with 0.05% Triton X-100 for 3 min.

After washing with PBS, cells were incubated with DuoLink blocking buffer at 37 °C for 30 min and were incubated with primary antibodies against EGFR (1:300, Abcam, ab52894) and ERBB2 (1:300, Abcam, ab16901) at 4 °C overnight. On the second day, cells were washed with washing buffer and then incubated with species-specific PLA probes for 1 h at 37 °C. Then, the ligation stock was diluted 1:5 in ddH_2_O to obtain a 1:40 dilution of the ligase, and this solution was then applied to the samples and incubated at 37 °C for 30 min. Samples were washed twice with washing buffer and polymerase diluted with a solution of amplification stock was added to amplify the fluorescence signal at 37 °C for 100 min. Finally, cells were covered with an anti-fluorescence quenching sealing liquid containing DAPI. Confocal microscopy (LSM 510, METALaser scanning microscope, Zeiss) was used to acquire images. Quantitative analysis of fluorescence intensity was performed with ImageJ.

### Co-immunoprecipitation (co-IP) assay

Protein was extracted following the protocol. Protein G Dynabeads (Invitrogen, USA) were cleaned and incubated with anti-EGFR (or anti-ERBB2) or control IgG for 20 min in PBST at room temperature. The beads were washed with PBST and incubated with total extracts under gentle shaking at room temperature for 1 h. The beads were washed, resuspended in 40 μl of 1 × loading buffer and boiled at 70 °C for 10 min. The samples were subjected to western blotting to detect the interaction between ERBB2 and EGFR.

### Mouse xenograft model

Athymic male nu/nu mice aged 6 weeks were used in this study. The experimental animals were purchased and housed at East China Normal University, and experiments were conducted with the approval of the Research Ethics Committee. Subcutaneous implant models were established by subcutaneously injecting 2 × 10^6^ cells stably expressing either sh-Ctrl, sh-GPAA1, Lv-Ctrl, or Lv-GPAA1. From the fifth day, each group of mice expressing Lv-Ctrl or Lv-GPAA1 was randomly divided into two groups, and the selected groups of Lv-Ctrl or Lv-GPAA1 mice were administered an injection of trastuzumab (MCE, HY-P9907, 20 mg/kg) or vehicle twice daily. Tumour size was monitored with callipers every 5 days. Tumour volume was calculated by the following formula: volume = 0.5 × length×width^2^. After 35 days, mice were sacrificed for tumour harvest, and tumour weights were measured. The obtained tumours were subjected to IHC staining to evaluate the expression of Ki-67 and p-AKT.

### Statistical analysis

The GraphPad 7.0 and SPSS 13.0 (SPSS, Chicago, IL, USA) software packages were used to perform statistical analyses. Cumulative survival curves were calculated using the Kaplan-Meier method, and differences among the groups were evaluated by the log-rank test. Comparisons between two groups were validated by two-tailed Student’s t-tests, and differences among three or more groups were evaluated by ANOVA. Two-way ANOVA was conducted to evaluate significant differences among two or more classification conditions. Univariate and multivariate Cox proportional hazards regression analyses were performed to clarify the factors influencing survival. All error bars in this study represent the mean ± S.D. Statistical significance is indicated as follows: *p* > .05 = ns, *p* < .05 = *, *p* < .01 = **, *p* < .001 = ***.

## Results

### Chromosomal amplification resulting in aberrantly up-regulated GPAA1 expression is correlated with poor prognosis in gastric cancer

**Fig. 1:**
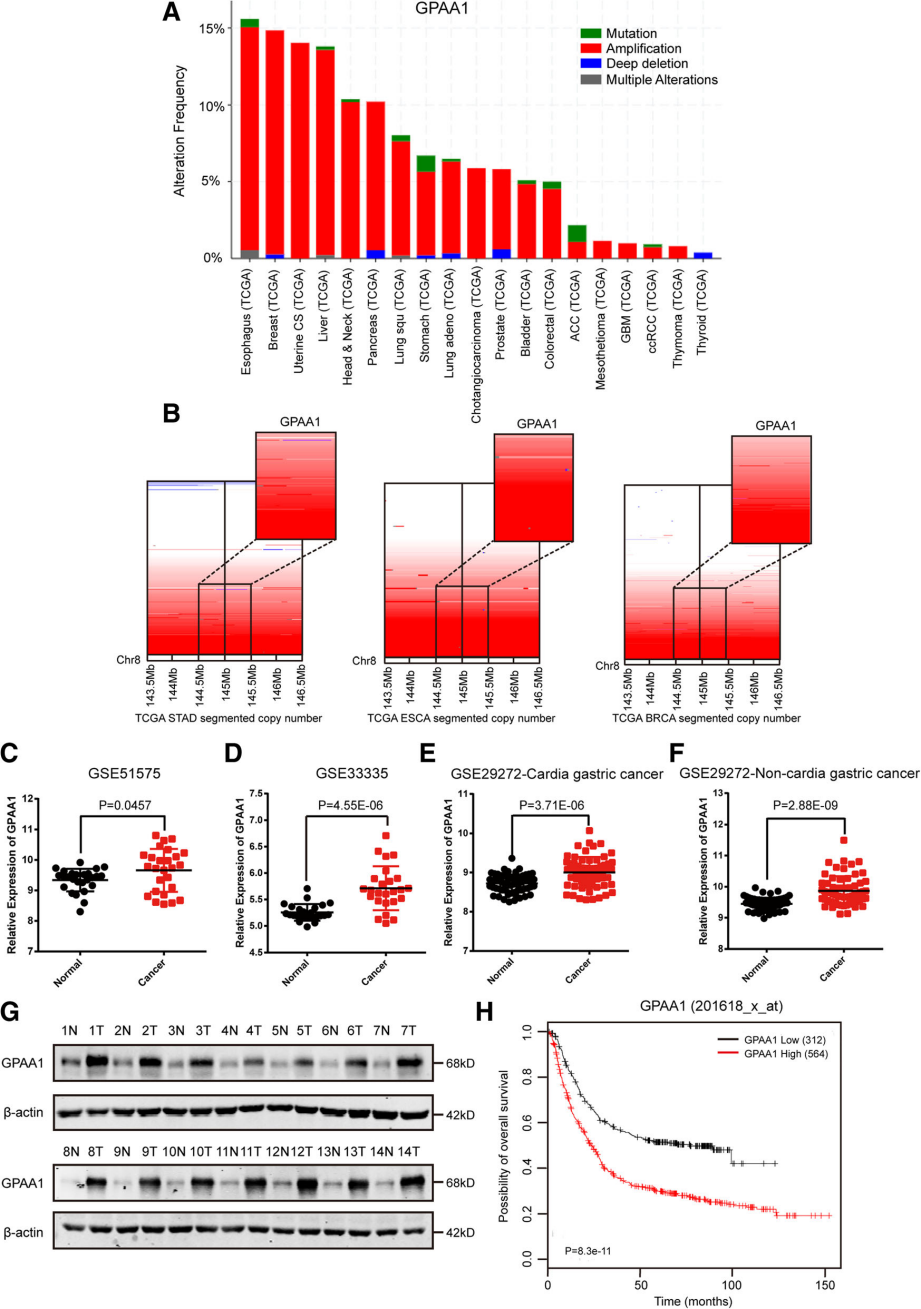
Chromosomal amplification resulting in aberrantly up-regulated GPAA1 expression is correlated with poor prognosis in gastric cancer. **a** Copy number analysis of TCGA samples of several types of cancers via the cBioPortal database. **b** Copy number analysis of GPAA1 in the TCGA stomach carcinoma, oesophageal carcinoma and breast carcinoma databases were performed via the UCSC Xena online tool. **c**-**f** GPAA1 expression analysis in tumours and normal tissues using three independent cohorts, (**c**, GSE51575, *n* = 26; **d**, GSE33335, *n* = 25; **e**, GSE29272, cardia gastric cancer samples, *n* = 62; and **f**, non-cardia gastric cancer samples, *n* = 72). **g** GPAA1 expression assessed in paired gastric cancer and normal tissues collected at Ren Ji Hospital, *n* = 14. **h** Kaplan-Meier analysis of the overall survival of GC patients according to GPAA1 mRNA expression, generated based on GEO cohorts

As previously mentioned, GPAA1 expression is extensively upregulated in liver cancer, breast cancer, head and neck cancer, and colorectal cancer. We sought to systematically explore cancer-related alterations in GPAA1 and the underlying mechanism of its upregulation in the pan-cancer landscape. Copy number analysis of samples from The Cancer Genome Atlas (TCGA) using the cBioPortal database indicated that GPAA1 expression was extensively upregulated in various types of malignancies, such as lung, pancreas, uterine, liver and prostate cancers (Fig. 1a), and was particularly upregulated in a considerable proportion of gastric cancers, oesophageal cancers and breast cancers (Fig. 1b). To further investigate the expression pattern of GPAA1 in gastric cancer, data mining was performed based on the GSE51575, GSE33335 and GSE29272 datasets (Fig. 1c, d, e and f) GPAA1 expression was significantly upregulated in cancer compared to that in the adjacent tumour. In addition, the noticeably increased expression of GPAA1 in gastric cancer was validated through paired tumour and normal tissues obtained from a sample library established at Ren Ji Hospital, School of Medicine, Shanghai Jiao Tong University (Fig. 1g). Moreover, the correlation between GPAA1 expression and the survival rate of gastric cancer patients was examined via the Kaplan-Meier method and log-rank tests based on several cohorts in the GEO database; higher GPAA1 expression predicted poorer survival (Fig. 1h). Thus, we concluded that GPAA1 is overexpressed in gastric cancer and is closely related to an unsatisfactory prognosis.

### GPAA1 promotes proliferation, colonization, and the G1-to-S phase transition in the cell cycle

**Fig. 2:**
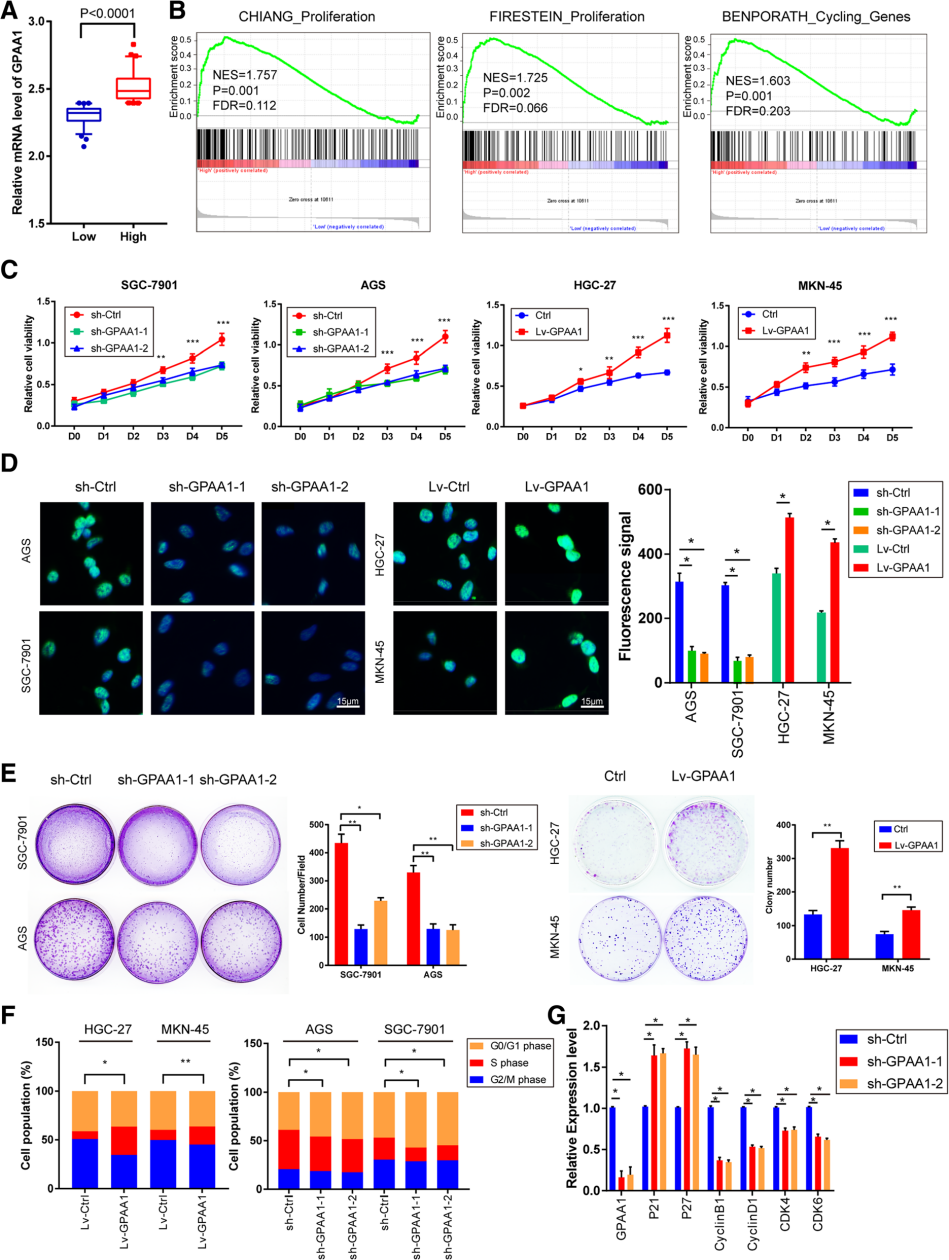
GPAA1 promotes proliferation, colonization, and the G1-to-S phase transition in the cell cycle. **a** GPAA1 expression levels in GPAA1-high samples compared with their low-GPAA1 counterparts among 300 GC patients in the GSE66229 cohort. **b** GSEA of specimens with high and low expression of GPAA1 based on the data from GES66229 (CHANG_Proliferation, NES = 1.757, *P* = 0.001, FDR = 0.112; FIRESTEIN_Proliferation, NES = 1.725, *P* = 0.002, FDR = 0.066; and BENPORATH_Cycling_Genes, NES = 1.803, *P* = 0.001, FDR = 0.203). **c** A CCK-8 assay was conducted using sh-Ctrl, sh-GPAA1–1, sh-GPAA1–2 (SGC-7901 and AGS), Lv-Ctrl, and Lv-GPAA1 cells (HGC-27 and MKN-45). **d** Immunofluorescence staining of Ki-67 in the GPAA1-upregulated, GPAA1-downregulated, and control groups. **e** Colony formation assay in the GPAA1-upregulated, GPAA1-downregulated, and control groups. **f** Cell cycle assay in the GPAA1-upregulated, GPAA1-downregulated, and control groups. **g** Gene expression analysis of GPAA1, P21, P27, cyclin B1, cyclin D1, CDK4, and CDK6 in the sh-Ctrl and sh-GPAA1 groups. *P < 0.05, **P < 0.01, ***P < 0.001

To elucidate the biological function of GPAA1 in gastric cancer, GSEA was performed based on the RNA sequencing (RNA-seq) data for 300 gastric cancer patients in the GSE66229 cohort. First, the expression profile of the samples was divided into the GPAA1-low group and GPAA1-high group based on the medium value according to the expression level of GPAA1 (Fig. 2a). GSEA analysis indicated that gene sets relevant to cell proliferation and the cell cycle were enriched in samples with high GPAA1 expression (Fig. 2b). To acquire further insight into whether GPAA1 could regulate cell proliferation and the cell cycle, we conducted a series gain-of-function and loss-of-function studies in gastric cancer cells. The GPAA1 expression pattern in gastric cancer cells was examined by western blotting and real-time PCR, which showed that the expression of GPAA1 in AGS and SGC-7901 cells was higher than that in the other cell lines (Additional file 1: Figure S1A and D). We selected AGS and SGC-7901 cells for stable knockdown and HGC-27 and MKN-45 cells for artificial upregulation of GPAA1 expression (Additional file 1: Figure S1B, C, E, F). Knockdown of GPAA1 expression significantly attenuated the proliferation of cancer cells, while GPAA1 overexpression greatly promoted growth (Fig. 2c). Additionally, the data showed that GPAA1 silencing contributed to an appreciable reduction in Ki-67 expression and that the overexpression of GPAA1 resulted in an opposite effect (Fig. 2d). Furthermore, the colonization of cancer cells was also positively regulated by GPAA1; GPAA1 silencing dramatically reduced the colonization ability, while GPAA1 upregulation profoundly promoted this ability (Fig. 2e). Flow cytometric analysis revealed that upregulation of GPAA1 profoundly suppressed but knockdown of GPAA1 increased the proportion of cells in the G0/G1 phase, indicating that GPAA1 may stimulate the G1-to-S phase transition in stomach cancer cell (Fig. 2f,and Additional file 2: Figure S2). In addition, the real-time PCR assay showed that the expression of cell cycle inhibitory proteins (P21 and P27) was sharply increased in GPAA1-silenced cells, while that of cell cycle promoters (cyclin B1, cyclin D1, CDK4, and CDK6) decreased (Fig. 2g). Similar results were observed in GPAA1-overexpressing cells (Additional file 1: Figure S1G). In conclusion, GPAA1 can positively regulate proliferation, colonization, and the G1-to-S transition in the cell cycle.

### GPAA1 promotes the metastasis and invasion of gastric cancer

**Fig. 3:**
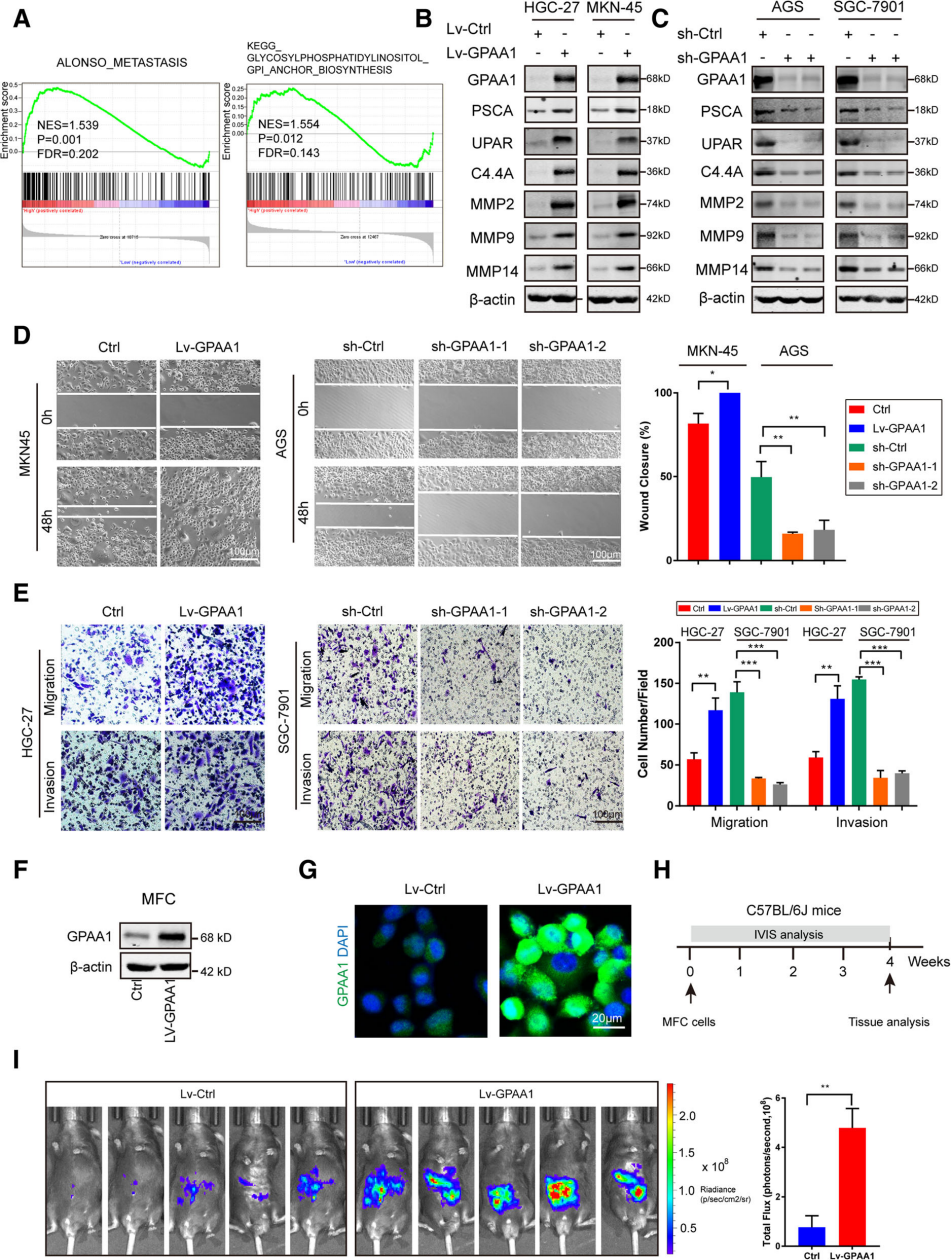
GPAA1 promotes the metastasis and invasion of gastric cancer. **a** GSEA of specimens with high and low expression of GPAA1 based on the data from GES66229 (ALONSO_METASTASIS, NES = 1.539, *P* = 0.001, FDR = 0.202; KEGG_GPI_ANCHOR_BIOSYNTHESIS, NES = 1.554, *P* = 0.012, FDR = 0.143. **b-c** WB analysis of PSCA, UPAR, C4.4A, MMP2, MMP9, and MMP14 in the GPAA1-upregulated, GPAA1-downregulated, and control groups. **d** Wound healing assays were conducted using GPAA1-knockdown, GPAA1-overexpressing and control AGS and MKN-45 cells (Scale bar: 100 μm), and the percentage of wound closure was calculated from the widths of the wound at 0 h and 48 h. The means ± SDs of three independent experiments are shown. **e** Transwell assays were performed with GPAA1-knockdown, GPAA1-overexpressing and control SGC-7901 and HGC-27 cells to assess the migration and invasive abilities (Scale bar: 50 μm). The number of cells migrating through the inserts was calculated, and the means ± SDs of three independent experiments are shown. **f** The overexpression efficiency of GPAA1 in MFC cells was tested by western blotting. **g** The overexpression efficiency of GPAA1 in MFC cells was assessed by immunofluorescence staining. **h** Experimental design of the in vivo study to explore the regulation of liver metastasis by GPAA1 in mice. **i** Liver metastasis in the Lv-Ctrl and Lv-GPAA1 groups was verified via the IVIS system and quantified by the total flux. *P < 0.05, **P < 0.01, ***P < 0.001

A prevailing view holds that some GPI-APs, such as PSCA [22], the urokinase receptor (u-PAR) [23], C4.4A [24], and MMPs [25–27], are deeply involved in cancer invasion and metastasis. Given that GPAA1 plays a vital role in GPI-AP synthesis, we hypothesized that GPAA1 may regulate cancer metastasis through altering the expression of specific GPI-APs. The GSEA analysis also showed that high expression of GPAA1 was closely related to metastasis and GPI-anchor synthesis (Fig. 3a). Indeed, GPAA1 positively modulated the expression of metastasis-associated GPI-APs (Fig. 3b and c). Next, wound healing and Transwell assays were conducted to evaluate the metastatic and invasive abilities of cancer cells in vitro after altering the expression of GPAA1. As shown in Fig. 3d and Fig. 3e, gastric cancer cells with GPAA1 knockdown exhibited significantly reduced migration and invasive abilities compared with those of control cells, consistent with the phenotype observed in GPAA1-overexpressing cells. To comprehensively investigate the function of GPAA1 in vivo, GPAA1 was overexpressed in MFC cells (Fig. 3f and g), and an animal model of liver metastasis was established (Fig. 3h). Upregulation of GPAA1 significantly promoted liver metastasis (Fig. 3i) and enhanced the expression of metastasis-related GPI-APs (Additional file 3: Figure S3A and B). Taken together, these findings indicate that GPAA1 accelerates the invasion and metastasis of gastric cancer by regulating the expression of GPI-APs, which perform crucial functions in metastasis.

### GPAA1 modulates EFGR-ERBB2 dimerization through influencing lipid raft formation

**Fig. 4:**
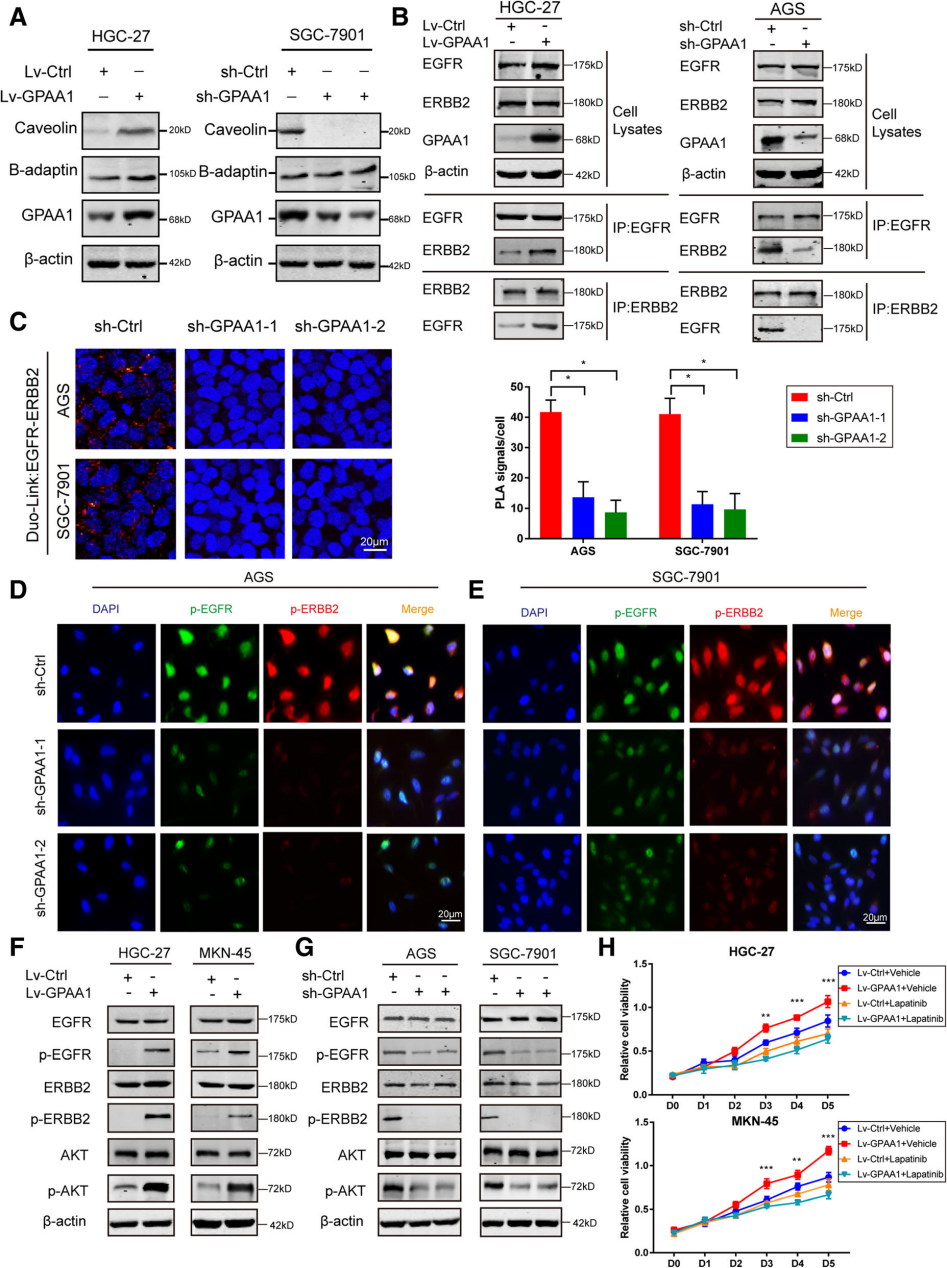
GPAA1 modulates EFGR-ERBB2 dimerization through influencing lipid raft formation. **a** Expression of caveolin (a marker of lipid rafts), B-adaptin (a marker of non-lipid rafts) and GPAA1 in GPAA1-knockdown, GPAA1-overexpressing and control SGC-7901 and HGC-27 cells was examined by western blotting. **b** A co-IP assay was performed to examine the EGFR-ERBB2 interaction in the GPAA1-knockdown, GPAA1-overexpressing and control groups. **c** The interaction of EGFR with ERBB2 was detected in the sh-Ctrl and sh-GPAA1 groups by an in situ PLA (red dots; *n* = 3). **d**-**e** Co-localization of EGFR and ERBB2 in the sh-ctrl and sh-GPAA1 groups was confirmed by immunofluorescence staining. **f-g** Expression of EGFR, p-EGFR, ERBB2, p-ERBB2, AKT, p-AKT, and β-actin in the GPAA1-knockdown, GPAA1-overexpressing and control groups was verified by western blotting. **h** A CCK-8 assay was conducted to evaluate the effect of lapatinib on GPAA1-overexpressing gastric cells. *P < 0.05, **P < 0.01, ***P < 0.001

GPI-APs are responsible for the formation and functional maintenance of lipid rafts, dynamic membrane domains consisting of cholesterol, sphingolipids, and many specific proteins that provide platforms for molecular trafficking and signal transduction. Because GPAA1 is essential to the synthesis and membrane binding of large-scale GPI-APs and eventually regulates lipid raft formation, we assessed the expression of lipid raft markers after altering GPAA1 protein levels. As shown in Fig. 4a, the expression of caveolin (a marker of lipid nanodomains) was notably increased or decreased after GPAA1 upregulation or suppression, respectively, compared with that in the control group, while the expression of B-adaptin (a non-lipid nanodomain marker) was unchanged. The HER2 proto-oncogene, named HER2/neu or (C-)ERBB2, was found to have upregulated expression in gastric cancer and to be closely associated with poor survival in patients with advanced gastric carcinoma [28]. The activation of HER2 and downstream signalling was found to be highly dependent on dimerization with EGFR [29, 30], preferentially localized and enriched in lipid rafts [31–33], a phenomenon that explained the robust efficacy of the dual EGFR-ERBB2 inhibitor lapatinib in malignant gastric cancer [34, 35]. Therefore, we performed co-IP to explore whether the interaction between EGFR and ERBB2 could be regulated by GPAA1. Intriguingly, GPAA1 overexpression noticeably strengthened but GPAA1 knockdown greatly impaired the EGFR-ERBB2 interaction (Fig. 4b). This result was validated via the DuoLink assay (Fig. 4c). Moreover, the confocal signal of p-ERFR and p-ERBB2 were weakened after GPAA1 downregulation (Fig. 4d and e). Furthermore, EGFR and ERBB2 phosphorylation, as well as downstream Akt activation, was modulated by the upregulation or downregulation GPAA1 expression (Fig. 4f and g). Accordingly, overexpression of GPAA1 profoundly promoted the proliferation of gastric cancer cells, an effect that was counteracted by the administration of trastuzumab, a blocker simultaneously targeting EGFR and ERBB2 (Fig. 4h). Furthermore, Lapatinib also exerted powerfully inhibitory effect on proliferation of gastric cancer cells, AGS and SGC-7901, which endogenously generate high expression of GPAA1 (Additional file 4: Figure S4). Therefore, we concluded that GPAA1 promotes the interaction between EGFR and ERBB2 and the signalling of the downstream promoter of proliferation—Akt—to enhance the uncontrolled growth of gastric cancer cells.

### GPAA1 facilitates in vivo tumour growth, which can be inhibited by trastuzumab

**Fig. 5:**
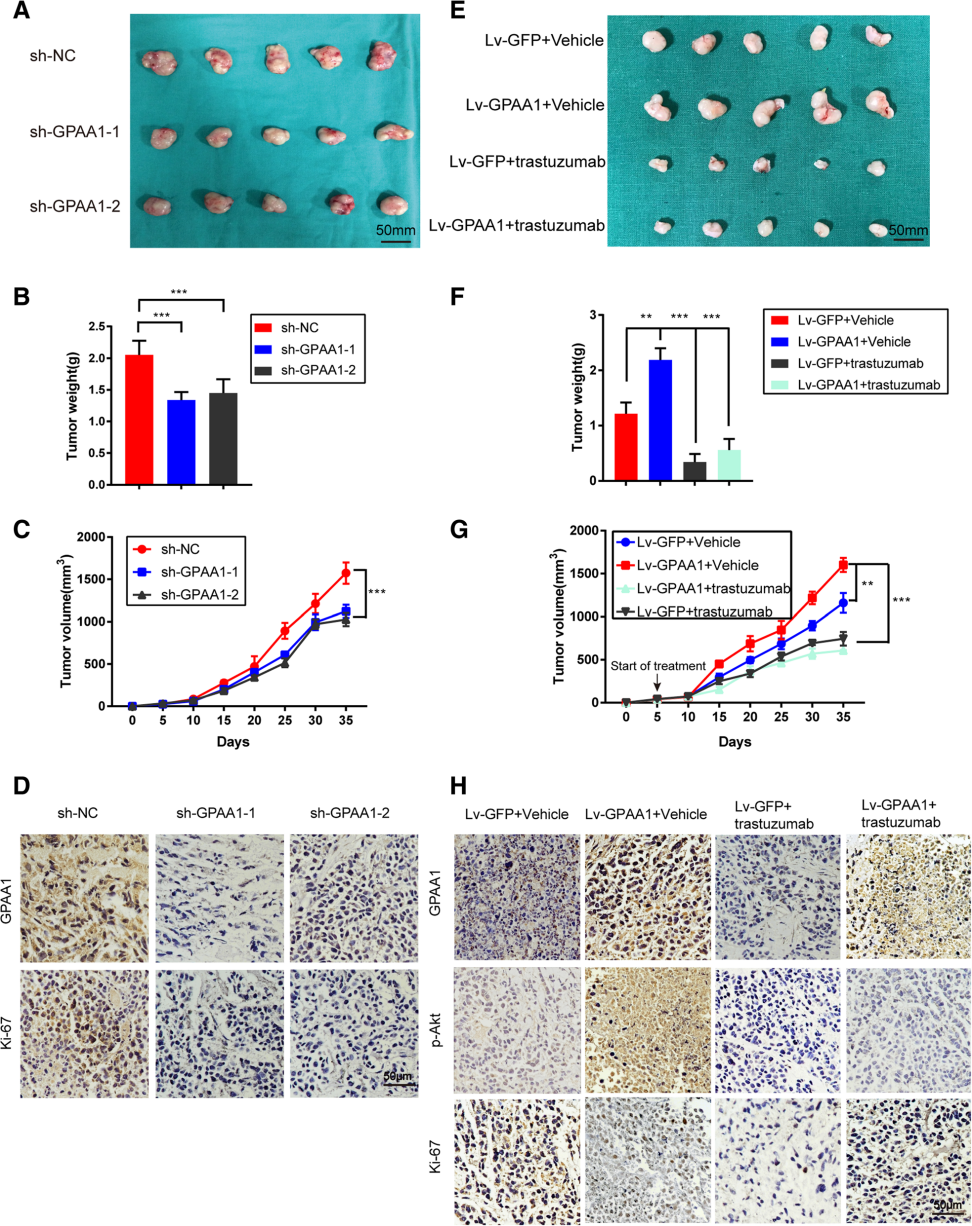
GPAA1 facilitates in vivo tumour growth, which can be inhibited by trastuzumab. **a** Tumours isolated from mice injected subcutaneously with AGS cells (sh-NC, sh-GPAA1–1, sh-GPAA1–2). **b**-**c** Tumour weights and tumour volumes in the GPAA1-Ctrl and GPAA1-downregulation groups. **d** IHC staining of GPAA1 and Ki-67 in sh-Ctrl and sh-GPAA1 tumours (scale bar: 50 μm). **e** Tumours obtained from mice injected subcutaneously with HGC-27 cells with either over- or normal expression of GPAA1 and treated with vehicle or trastuzumab. **f-g** Tumour weights and tumour volumes in each group. **h** IHC staining of GPAA1, p-AKT and Ki-67 in each group (scale bar: 50 μm). *P < 0.05, **P < 0.01, ***P < 0.001

To identify the biological function of GPAA1 in vivo, we established a subcutaneous tumour model. Suppression of GPAA1 attenuated tumour growth in vivo, as exhibited by the tumour weight and tumour volume measurements (Fig. 5a, b and c). IHC staining showed that expression of Ki-67, a proliferation marker, was also disrupted after knockdown of GPAA1 expression (Fig. 5d). However, overexpression of GPAA1 dramatically promoted tumour growth and Akt activation, which were reversed by trastuzumab treatment (Fig. 5e, f, g and h), indicating the diagnostic and therapeutic potential of GPAA1 in gastric cancer.

### Expression pattern and clinical value of GPAA1 in human gastric cancer

**Fig. 6:**
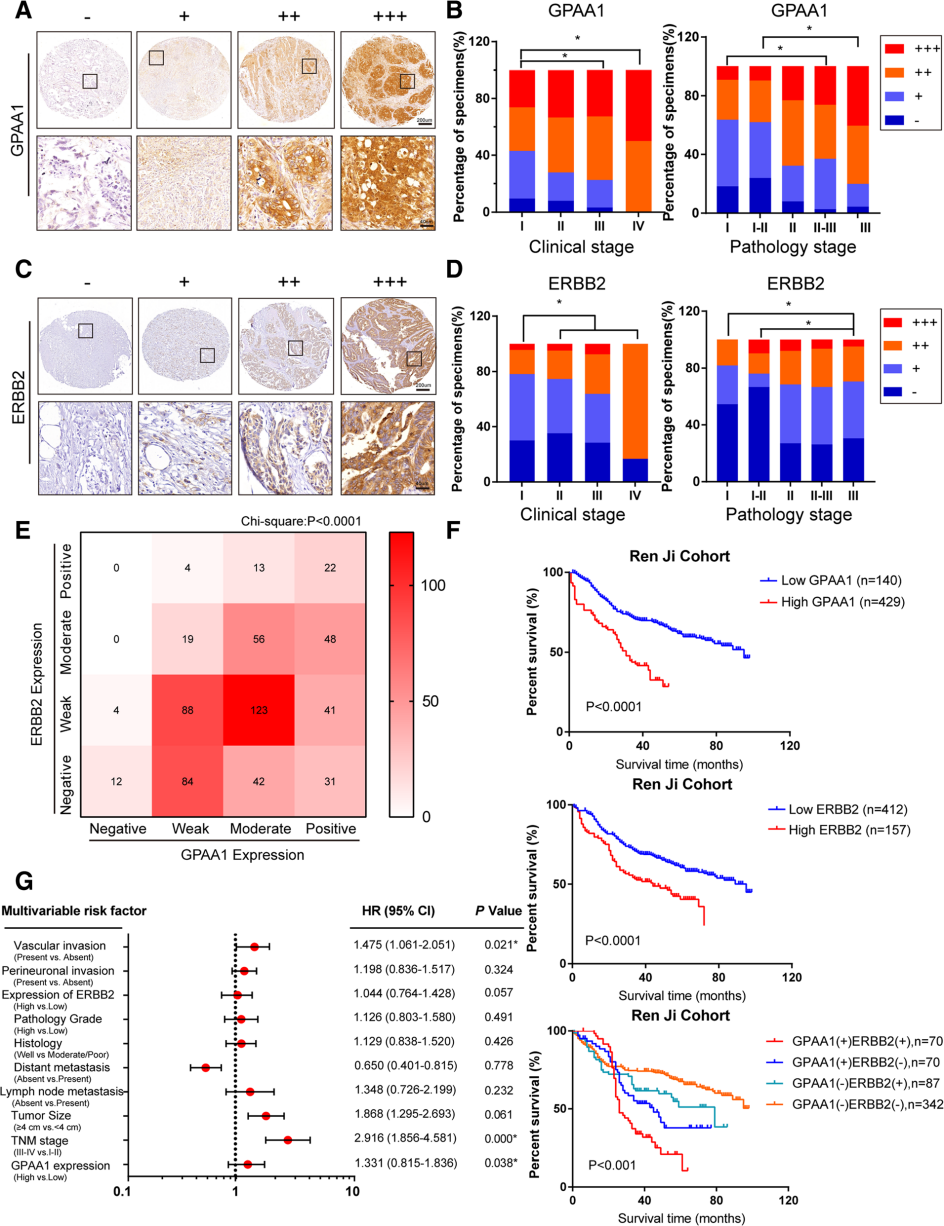
Expression pattern and clinical value of GPAA1 in human gastric cancer. **a** Representative IHC staining for GPAA1 from the Ren Ji cohort, which contained 587 GC patients. The expression level was scored as -, +, ++, or +++ (Scale bars: 200 μm and 40 μm). **b** Percentage of specimens with different expression levels of GPAA1 according to clinical stage and pathological stage (569 patients with collected prognostic information were enrolled). **c** Representative IHC staining of ERBB2 from the Ren Ji cohort, which contained 587 GC patients. **d** Percentage of specimens with different expression levels of ERBB2 according to clinical stage and pathological stage (569 patients). **e** Correlation between GPAA1 and ERBB2 expression in the Ren Ji cohort (587 patients, Chi-square: *P* < 0.0001, log odds ratio: 4.794 (2.964–7.755), *P* < 0.0001). **f** Kaplan-Meier curve for the prognosis of patients with high and low levels of GPAA1 or ERBB2 expression in the Ren Ji cohort (569 patients). The study involved patients who had received gastrectomy from January 2006 to December 2011, and the final follow-up date was December 31, 2017. **g** Multivariate Cox regression analysis of the Ren Ji cohort (569 patients). *P < 0.05, **P < 0.01, ***P < 0.001

Next, we investigated the expression profile of GPAA1 in gastric cancer and the relationship between GPAA1 expression and prognosis. A set of tissue microarrays containing 587 pathologist-confirmed specimens was subjected to IHC staining, and 569 of these clinically annotated specimens were selected for prognostic analysis. The expression level of GPAA1 was defined as -, +, ++, or +++ with respect to the staining area and intensity; representative images are shown in Fig. 6a. Interestingly, the expression of GPAA1 gradually increased with increasing clinical stage and pathological stage (Fig. 6b). Furthermore, we explored the expression pattern and clinical relevance of ERBB2. Consistent with previous reports, ERBB2 expression was strongly upregulated in a stepwise manner with the progression of clinical stage and pathological stage (Fig. 6c and d). Intriguingly, correlation analysis indicated that expression of GPAA1 was highly related to that of ERBB2 (Fig. 6e). Then, the Kaplan-Meier method and log-rank test were used to evaluate the effects of GPAA1 and ERBB2 alone and in combination on the survival rate of gastric cancer patients. The results demonstrated that patients with lower expression of GPAA1 or ERBB2 lived longer than their counterparts with higher expression of these proteins. In addition, analysis of the combined effects of GPAA1 and ERBB2 showed that GPAA1-ERBB2-high patients exhibited the worst overall survival rates, while patients with GPAA1-ERBB2-low expression lived longest (Fig. 6f). In addition, univariate and multivariate analyses utilizing a Cox proportional hazards model were conducted to investigate the relationship between GPAA1 expression and patient outcomes. GPAA1 expression, ERBB2 expression, age, tumour size, vascular invasion, TNM stage, lymphatic metastasis, and perineuronal invasion were significantly correlated with overall survival (Table 2). In multivariate Cox regression analysis, GPAA1 expression, TNM stage, and vascular invasion were independent predictors of poor prognosis (Fig. 6g). In summary, GPAA1 expression was highly synergistic with ERBB2 expression, and this expression was correlated with the progression of gastric cancer and predicted unsatisfactory outcomes.

**Table 2:** Univariate analysis of prognostic parameters for survival in patients with GC

Prognostic parameter	HR	95% CI	*P* value
Expression of GPAA1 (High vs Low)	0.735	0.530–1.019	0.045
Age (≥65 vs. < 65)	1.486	1.142–1.932	0.003
Gender (Male vs. Female)	0.871	0.662–1.147	0.326
TNM Stage (III-IV vs. I-II)	4.754	3.487–6.482	0.000
Tumor Size (≥4 cm vs. < 4 cm)	3.192	2.274–4.481	0.000
Pathology Grade (I, I-II, II vs. II-III, III)	1.288	0.961–1.725	0.090
Expression of ERBB2 (High vs Low)	1.137	0.855–1.511	0.003
Lymphatic metastasis (Present vs. Absent)	3.852	2.751–5.934	0.000
Distant metastasis (Present vs. Absent)	1.176	0.165–8.395	0.871
Vascular invasion (Present vs. Absent)	2.435	1.795–3.303	0.000
Perineuronal invasion (Present vs. Absent)	2.152	1.531–3.025	0.000
Histology (Poor vs. well & moderate)	0.833	0.638–1.807	0.179

*HR* Hazard ratio, *CI* Confidence interval

## Discussion

In the present study, we comprehensively confirmed that GPAA1 expression was extensively upregulated in malignant tumours and offered the first demonstration of the expression pattern, biological function, and underlying mechanism of GPAA1 in gastric cancer. In summary, the GPI transamidase complex component GPAA1 was validated to be overexpressed in gastric cancer and to exert oncogenic effects, including the acceleration of proliferation and metastasis. The main points in this study are summarized as follows: 1) chromosomal amplification of GPAA1 genetically promoted its upregulation in gastric cancer and its positive correlation with poor survival; 2) GPAA1 facilitated the proliferation of and the G1-to-S phase transition in gastric cancer cells; 3) through upregulating the expression of metastasis-promoting GPI-APs, GPAA1 appreciably accelerated invasion and metastasis both in vitro and in vivo; 4) GPAA1 promoted EGFR-ERBB2 interaction and downstream signalling, which activated proliferation via enhancing lipid raft stabilization; 5) GPAA1 silencing inhibited tumour growth, and the dual EGFR-ERBB2 inhibitor lapatinib successfully reversed tumour growth promoted by GPAA1 overexpression; and 6) GPAA1 expression was correlated with ERBB2 expression and predicted unfavourable patient outcomes (Fig. 6). From these results, we can deduce that GPAA1 could be a promising diagnostic biomarker and potential therapeutic target for gastric cancer. For the diagnosis, upregulation of GPAA1 is closely associated with poor prognosis in gastric cancer, and its expression is positively related ERBB2. Hence, the examination of GPAA1 expression is a potential approach for prognostic prediction. For the treatment, the research of oncogenic function indicated that GPAA1 can greatly promote tumour growth and metastasis, so destroy the effect of GPAA1 will possibly prevent tumour progression. Moreover, the results showed that GPAA1 promoted tumour growth via the enhancement of ERBB signalling, which provided a strategy of combined therapy with ERBB inhibitors or antibodies in gastric cancer.

Accumulating evidence suggests that GPI-APs are involved in carcinogenesis and the progression of multiple malignant tumours. Collectively, the promotive function exerted by GPI-APs in cancer can be classified as direct or indirect. On the one hand, several kinds of GPI-APs, such as CEA, PSA, MMPs, CD46, CD55, and CD59, directly contribute to proliferation, metastasis, and immune escape; on the other hand, GPI-APs participate in the establishment and formation of lipid rafts, platforms suitable for numerous protein interactions and signal mediation [36–40]. Therefore, inhibiting the upregulation or activity of cancer-associated GPI-APs is a promising method for targeted therapy.

Approaches to suppress the activity of GPI-APs are currently available, one of which is the cleavage of GPI anchors from the attached proteins. The physiological function of GPI-APs depends heavily on structural integration, and if the ligation between the attached protein and GPI anchor is destroyed, GPI-APs are released from the membrane and lose their function. For instance, GPI anchorless uPAR was soluble and could eliminate uPA, resulting in the inhibition of metastasis [41]. In addition, cancer cells lacking GPI-attached CEA could induce anoikis [42]. For instance, glycosylphosphatidylinositol-specific phospholipase D (GPI-PLD) can hydrolyse and release GPI anchors in humans [43]. Upregulation of endogenous GPI-PLD was reported to induce spontaneous CEA release in colon cancer cells [44]. In addition, the angiotensin-converting enzyme (ACE) is a key regulator of blood pressure, which also has an activity of releasing glycosylphosphatidylinositol (GPI)-anchored protein. ACE can shed various kinds of GPI-APs from the cell surface via the cleavage site at the mannose-mannose linkage [45]. Besides, some phosphodiesterase, such as GDE2, also exerts its function of incising GPI-anchored proteins at the plasma membrane [46]. Unfortunately, the strategy of overexpressing these enzymes for cancer therapy is not realistic, not only because of technical difficulties in constantly heterogenous expression of specific genes in human, but also because of the toxicity of widespread shedding of GPI anchors in the human body. However, suppressing the process of GPI-AP synthesis controlled by GPAA1 is a promising tactic in cancer therapy. First, the function of transferring GPI to its attached protein is extraordinarily important in GPI-AP synthesis; second, repressing the enzymatic activity of aberrantly overexpressed GPAA1 by inhibitors or antibodies is easier and more feasible than other approaches, such as overexpressing enzymes which cause the cleavage of GPI-APs, or designing antibodies or inhibitors specifically targeting one type of GPI-AP. Furthermore, in addition to use as a monotherapy, a GPAA1 inhibitor could be adopted as a combined therapy with trastuzumab, given that GPAA1 regulates the ERBB2 phosphorylation and downstream pathway activation. Until now, there is not existed any developed or commercialized inhibitor of GPAA1, but the development should be carried forward in the future.

Unanswered questions remain that require further exploration of GPAA1- and GPI-AP-related enzymes and proteins in gastric cancer. First, the expression patterns and biological functions of the GPI transamidase complex components in addition to GPAA1—PIG-T, GPI8, PIG-S, and PIG-U—are unknown in gastric cancer. Second, due to the major role of GPAA1 in GPI-AP synthesis, the toxicity and side effects of GPAA1 inhibitors deserve in-depth study. Third, additional oncoproteins and oncogenic signalling pathways modulated by GPAA1 need to be identified, which is critical knowledge for studies of chemoresistance and combination therapy.

## Conclusion

**Fig. 7:**
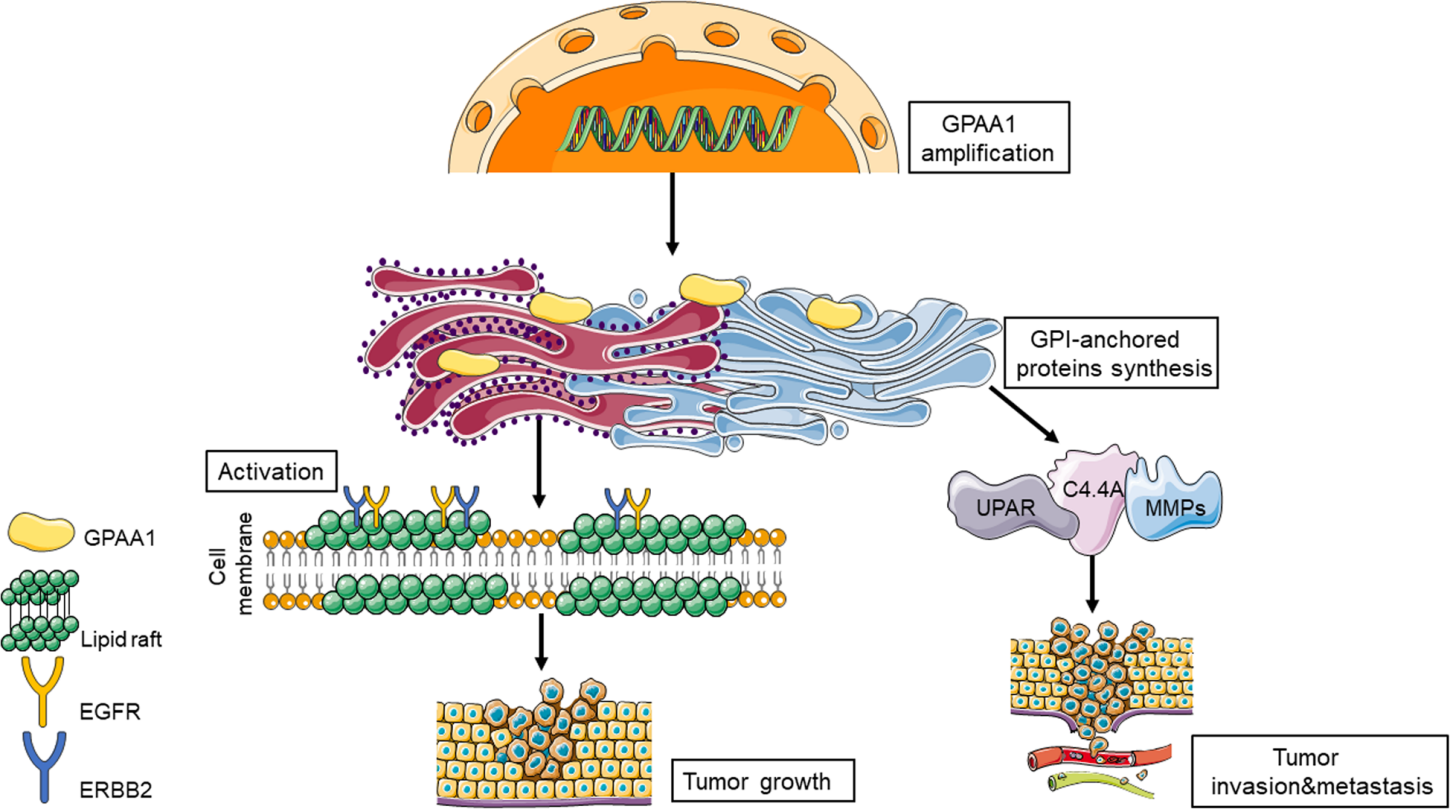
Proposed model for GPAA1 promoting growth and metastasis of gastric cancer. The genetically amplification results in upregulation of GPAA1 in gastric cancer, which consequently promotes growth and metastasis of this malignant tumour. On the one hand, the upregulation of GPAA1 facilitates increased amount of metastasis-promotive GPI-anchored protein, such as MMPs, C4.4A, and UPAR; on the other hand, the aberrant overexpression of GPAA1 enhances the formation of lipid raft, which next activates ERBB signalling to promote proliferation of gastric cancer

In conclusion, our research showed that GPAA1 is significantly overexpressed in gastric cancer due to chromosomal amplification and performs significant functions in malignant transformation and progression, including mediation of tumour growth and metastasis. In addition, GPAA1 regulates the interaction between EGFR and ERBB2 and stimulates downstream signal transduction to promote proliferation (Fig. 7). Moreover, the expression of GPAA1 is independently correlated with poor survival and outcomes of gastric cancer patients. These results indicate that GPAA1 is a potential diagnostic biomarker and therapeutic target for gastric cancer as a monotherapy or in combination with trastuzumab or other drugs.

## Additional files

Additional file 1: **Figure S1.** GPAA1 expression in GC cell lines and verification of knockdown and overexpression efficiency. (A, D) GPAA1 expression in GES-1, MGC-803, AGS, BGC-823, MKN-45, NCI-N87, HGC-27, and SGC-7901 cells at the protein and mRNA levels. (B, E) Knockdown efficiency in AGS and SGC-7901 cells at the protein and mRNA levels. (C, F) Overexpression efficiency in HGC-27 and MKN-45 cells at the protein and mRNA levels. (G) Gene expression analysis of GPAA1, P21, P27, cyclin B1, cyclin D1, CDK4, and CDK6 in the Lv-Ctrl and Lv-GPAA1 groups. *P < 0.05, **P < 0.01, ***P < 0.001. (TIF 8883 kb)

Additional file 2: **Figure S2.** Verification of cell cycle assay by flow cytometry. (A, B) Cell cycle analysis in AGS and SGC-7901 cells transfected with sh-Ctrl, sh-GPAA1–1, and sh-GPAA1–2. (C, D) Cell cycle analysis in HGC-27 and MKN-45 cells transfected with Lv-Ctrl and Lv-GPAA1. (TIF 14230 kb)

Additional file 3: **Figure S3.** Haematoxylin and eosin (H&E) and IHC staining in liver metastasis models. (A) H&E and IHC staining of MMP2, MMP9, and UPAR in liver tissues from the Lv-Ctrl group. (B) H&E and IHC staining of MMP2, MMP9, and UPAR in liver tissues from the Lv-GPAA1 group. (TIF 12167 kb)

Additional file 4: **Figure S4.** Lapatinib significantly reduce proliferation of gastric cancer cell lines with high expression level of GPAA1. (A) A CCK-8 assay was performed to evaluate the effect of lapatinib on AGS. (B) CCK-8 assay was conducted to test the inhibitory effect of Lapatinib on SGC-7901. *P < 0.05, **P < 0.01, ***P < 0.001. (TIF 2236 kb)

## Abbreviations

ALP: Alkaline phosphatase
CEA: Carcinoembryonic antigen
EGFR: Epidermal growth factor receptor
ERBB2: Erb-B2 receptor tyrosine kinase 2
GEO: Gene Expression Omnibus
GPAA1: GPI anchor attachment 1
GPC3: Glypican-3
GPI-Aps: Glycosylphosphatidylinositol (GPI)-anchored proteins
GPI-PLD: Glycosylphosphatidylinositol-specific phospholipase D
GPIT: GPI transamidase complex
GSEA: Gene set enrichment analysis
PSCA: Prostate stem cell antigen
TCGA: The Cancer Genome Atlas
TKI: Tyrosine kinase inhibitor
u-PAR: Urokinase receptor
VEGFR: Vascular endothelial growth factor receptor
