# Supplementary figures and images for: GPAA1 promotes gastric cancer progression via upregulation of GPI-anchored protein and enhancement of ERBB signalling pathway
Source: J Exp Clin Cancer Res. 2019 May 22;38:214. doi: 10.1186/s13046-019-1218-8 (PMC6532258; doi:10.1186/s13046-019-1218-8)

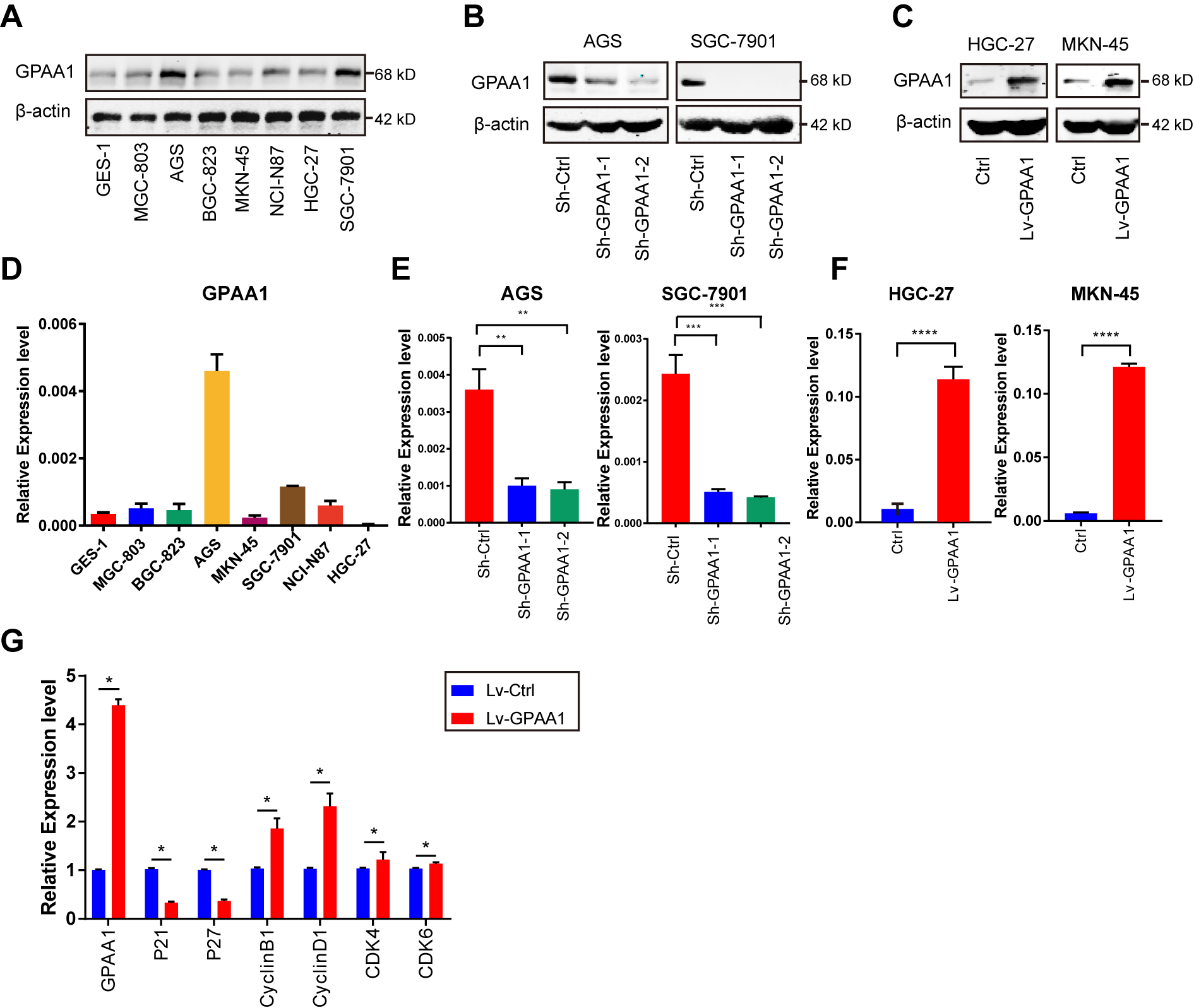

Supplement: Supplementary file 1 — Figure S1. GPAA1 expression in GC cell lines and verification of knockdown and overexpression efficiency. (A, D) GPAA1 expression in GES-1, MGC-803, AGS, BGC-823, MKN-45, NCI-N87, HGC-27, and SGC-7901 cells at the protein and mRNA levels. (B, E) Knockdown efficiency in AGS and SGC-7901 cells at the protein and mRNA levels. (C, F) Overexpression efficiency in HGC-27 and MKN-45 cells at the protein and mRNA levels. (G) Gene expression analysis of GPAA1, P21, P27, cyclin B1, cyclin D1, CDK4, and CDK6 in the Lv-Ctrl and Lv-GPAA1 groups. *P < 0.05, **P < 0.01, ***P < 0.001. (TIF 8883 kb) [file 13046_2019_1218_MOESM1_ESM.tif]

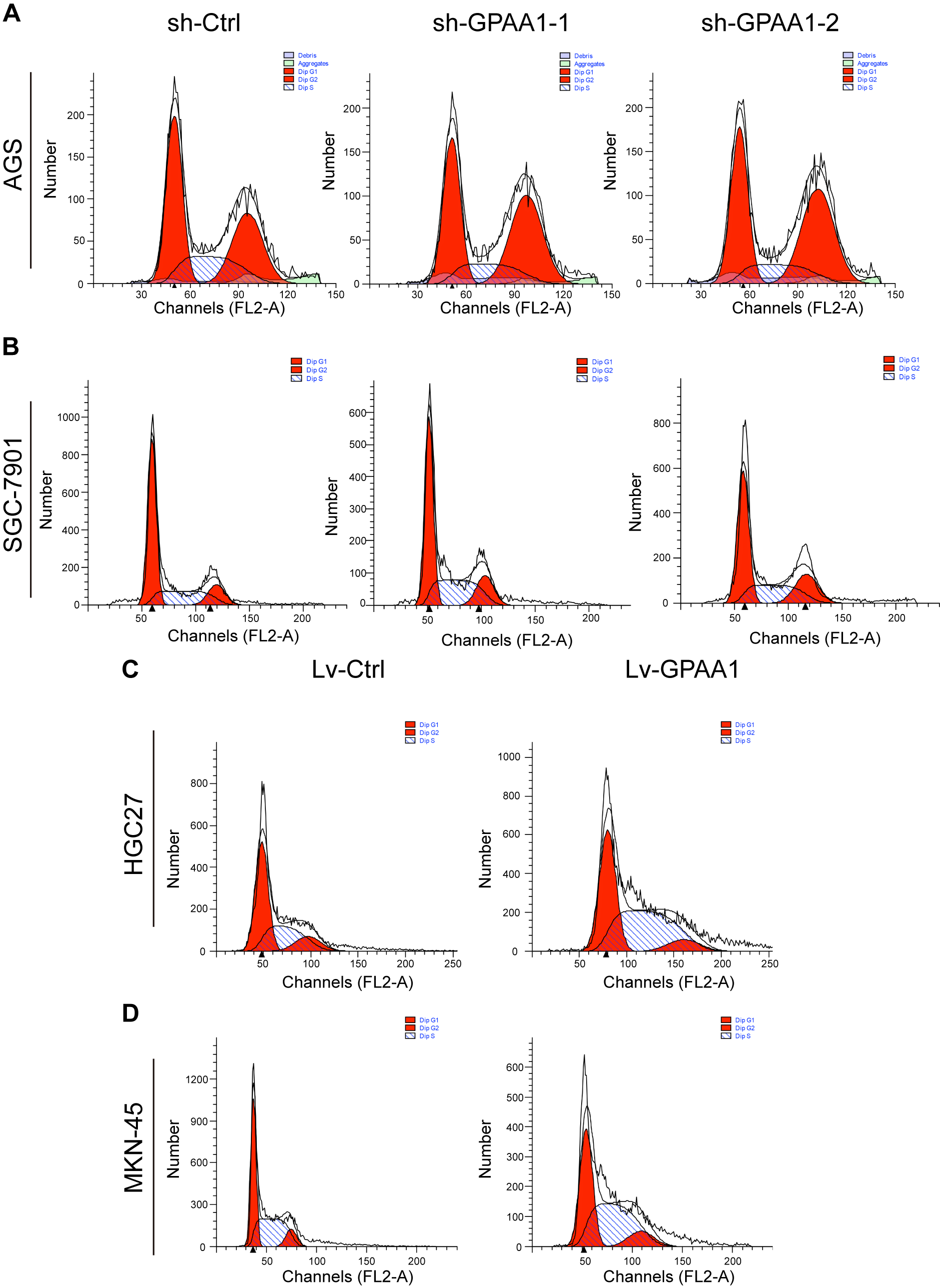

Supplement: Supplementary file 2 — Figure S2. Verification of cell cycle assay by flow cytometry. (A, B) Cell cycle analysis in AGS and SGC-7901 cells transfected with sh-Ctrl, sh-GPAA1–1, and sh-GPAA1–2. (C, D) Cell cycle analysis in HGC-27 and MKN-45 cells transfected with Lv-Ctrl and Lv-GPAA1. (TIF 14230 kb) [file 13046_2019_1218_MOESM2_ESM.tif]

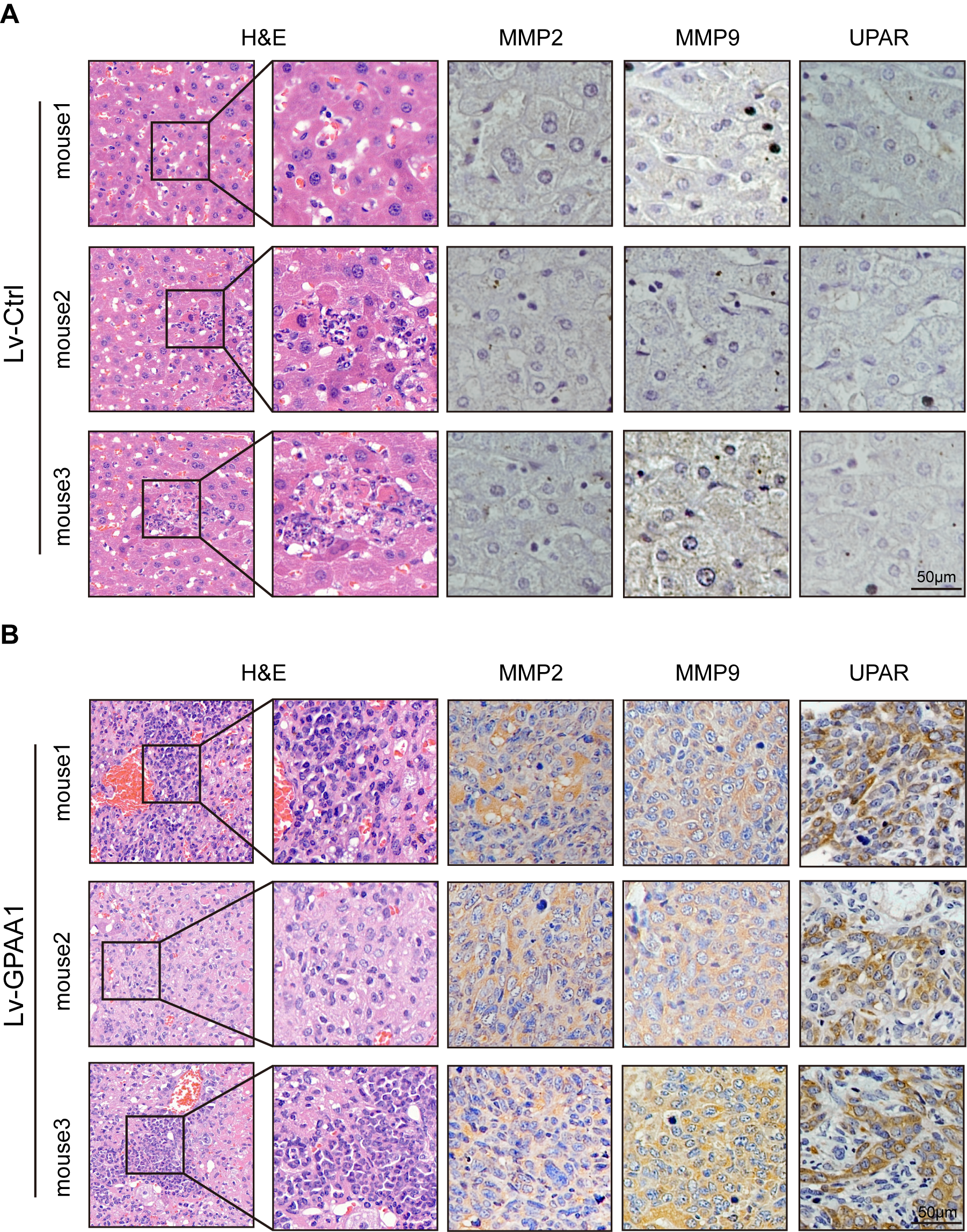

Supplement: Supplementary file 3 — Figure S3. Haematoxylin and eosin (H&E) and IHC staining in liver metastasis models. (A) H&E and IHC staining of MMP2, MMP9, and UPAR in liver tissues from the Lv-Ctrl group. (B) H&E and IHC staining of MMP2, MMP9, and UPAR in liver tissues from the Lv-GPAA1 group. (TIF 12167 kb) [file 13046_2019_1218_MOESM3_ESM.tif]

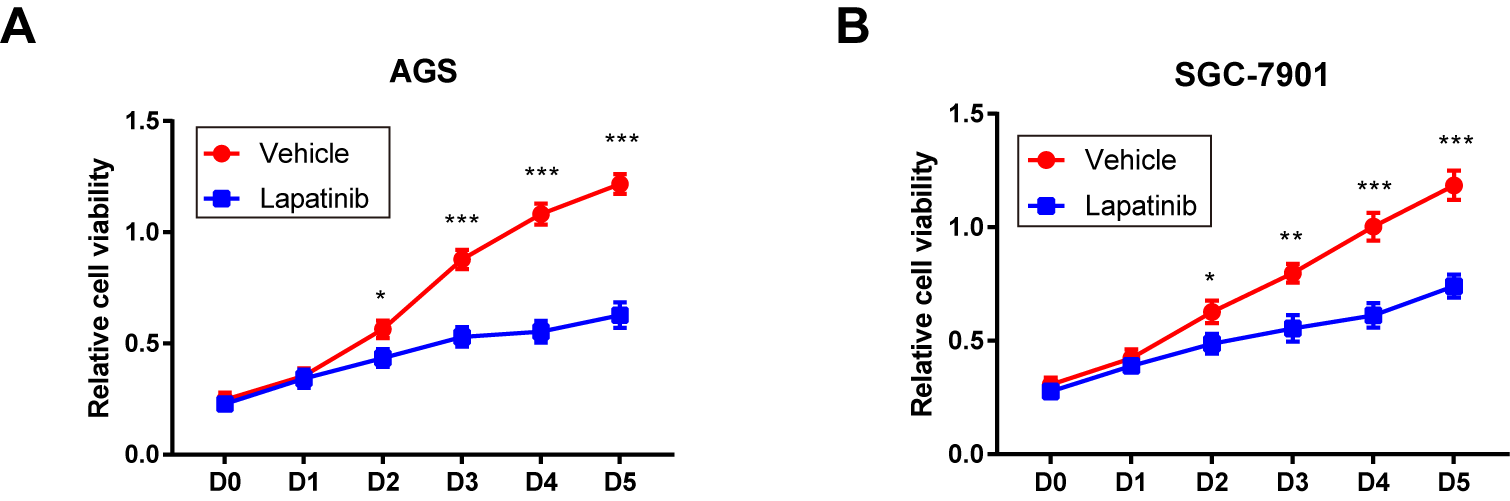

Supplement: Supplementary file 4 — Figure S4. Lapatinib significantly reduce proliferation of gastric cancer cell lines with high expression level of GPAA1. (A) A CCK-8 assay was performed to evaluate the effect of lapatinib on AGS. (B) CCK-8 assay was conducted to test the inhibitory effect of Lapatinib on SGC-7901. *P < 0.05, **P < 0.01, ***P < 0.001. (TIF 2236 kb) [file 13046_2019_1218_MOESM4_ESM.tif]
